# Animal-Assisted Intervention: A Promising Approach to Obesity Prevention for Youth With Autism Spectrum Disorder

**DOI:** 10.3389/fvets.2021.646081

**Published:** 2021-05-19

**Authors:** Aviva Must, Christina M. Mulé, Deborah E. Linder, Sean B. Cash, Sara C. Folta

**Affiliations:** ^1^Department of Public Health & Community Medicine, Tufts University School of Medicine, Boston, MA, United States; ^2^Division of Developmental and Behavioral Pediatrics, Department of Pediatrics, University of Rochester School of Medicine and Dentistry, Rochester, NY, United States; ^3^Division of Developmental and Behavioral Pediatrics, Department of Pediatrics, Tufts University School of Medicine, Boston, MA, United States; ^4^Tufts Institute for Human-Animal Interaction, Cummings School of Veterinary Medicine at Tufts University, North Grafton, MA, United States; ^5^Friedman School of Nutrition Science and Policy, Tufts University, Boston, MA, United States

**Keywords:** animal-assisted intervention, obesity, human-animal interaction, human-animal bond, autism spectrum disorder

## Introduction

In this opinion, we describe the justification for including companion dogs without special training (hereafter “pet dog”) within a family, as an animal-assisted approach to health promotion for children with autism spectrum disorder (ASD). ASD is a neurodevelopmental disorder that is characterized by persistent social impairments, verbal and non-verbal communication difficulties, and restrictive and repetitive patterns of behaviors, activities, and/or interests (1). A recent US CDC survey estimates 1 in 54 (1.85%) 8-year-old US children have been diagnosed with ASD (2). With obesity prevention as the key example, we review obesity risk factors in this population and demonstrate how a pet dog would, with appropriate considerations, contribute constructively to address the family-centered behavioral targets that encourage energy balance while also promoting health and wellness for the pet dog. The family environment represents a key venue for promoting heart healthy lifestyle behavior and one that is well-positioned for leveraging the human-animal bond. Food intake, physical activity, and sedentary behavior have been identified as the key drivers of excess weight gain in children and adults and are relevant for obesity prevention in youth with ASD as well as canine weight management and overall quality of life. Nonetheless, core features and several autism-associated characteristics present additional challenges and consideration when promoting behavior change for youth with ASD.

## Obesity in Children With ASD

The obesity epidemic has spared no population group, with a growing body of evidence that children and adolescents with ASD are at increased risk compared with their neurotypical peers (3, 4). Estimates from the National Survey of Children's Health (NSCH), a periodic nationally representative survey, indicate a 30–50% increased risk for obesity in children with ASD compared to their non-autistic counterparts (5, 6). The most recent NSCH (2016) estimated an obesity prevalence of 23% among children with ASD aged 10–17 years compared to 16% in children without ASD (7). Although addressing obesity is a pressing public health problem for all children, it is particularly so for children with ASD for whom secondary health issues that manifest in adulthood may limit their access to the least restrictive future living arrangement.

## Modifiable Obesity Risk Factors in Children With ASD

Obesity occurs when energy intake exceeds energy expenditure, net of the energy needed for growth. Both food intake and expenditure can be adversely impacted in children with ASD. Core features of ASD are impairments in social communication, language, and related cognitive skills and behavioral and emotional regulation and the presence of restricted, repetitive behaviors (1).

Addressing excess weight in a child with ASD must be considered in the context of its broad impact on family life. Parents and caregivers have many competing priorities beyond those of raising typically developing children. Children with ASD may attend school outside of the district. That additional travel, along with regular appointments with a range of specialists represent a heavy burden on families (8). These substantial demands, as well as meeting the needs of siblings and maintaining a “normal” family life, understandably may result in families placing a lower priority on healthy eating and physical activity patterns than might otherwise be the case (9, 10).

### Animal-Assisted Interventions

Maintaining a healthy weight is a challenge for many children and adolescents irrespective of the presence of a neurodevelopmental disorder. Few studies have directly examined the relationship between dog ownership and child obesity in the general population—and all are observational. One study of Australian children found a moderate significant protective effect of dog ownership on overweight for younger children but not older children (11). A second study from the UK found no association between dog ownership and child obesity cross-sectionally at age 7 or prospectively from age 7 to age 9 (12). Physical activity is the major pathway that would link dog ownership to obesity. A meta-analysis of dog ownership and adult obesity found that dog owners engage in significantly more walking and physical activity than non-owners. Although the presence of a pet dog in a household does not insure engagement, children in households with a dog are significantly more likely to meet physical activity guidelines (13). To date, there have been no randomized controlled intervention studies designed to assess the impact of dog ownership on child weight status or physical activity; the PLAYCE PAWS trial, launched in 2020, will directly test this hypothesis in a pilot exploratory study (14).

Prevention approaches that have been successful in typically developing children may not be similarly successful in youth with ASD. Nonetheless, inclusion of animals in obesity prevention efforts represents a promising approach that considers many of the unique features of ASD. Animals are increasingly a part of therapy with children with ASD with a positive impact on social and emotional functioning (15, 16). Dogs, in particular, provide relatively simple social and behavioral cues that children with ASD are better able to interpret (17).

AAI in children with ASD represents an emerging approach that has potential for effective obesity prevention. A systematic review and meta-analysis in 2018 of AAI in children with ASD provides a useful summary of studies to date that employed an experimental design (15). However, studies that incorporated dogs are quite limited and rarely include pet dogs in the home, favoring interventions with trained therapy or service dogs. Small effects on social interaction were identified in two dog-assisted play therapy pilot studies conducted in Hong Kong (18, 19). In all of the studies, the AAI was feasible and acceptable to participants. It should be noted that preventive obesity interventions require very large sample sizes to demonstrate significant results, given that obesity arises due to a small, but cumulative, positive energy balance. Demonstrating significant results in an AAI intervention for this population would likely require a sample size greater than would be feasible to enroll. Given mounting evidence that AAI can improve sustained focus, emotional stability, attitudes toward learning, and progress on individual goals, its utility for establishing and maintaining positive lifestyle behavior changes holds promise (20, 21).

## Discussion

### Potential Role for Pet Dogs in Promoting Healthy Behaviors

Although much of the evidence on AAI among children with ASD reflects the use of registered therapy animals or service animals, pet dogs offer several significant potential advantages over therapy dogs. It is not always possible for families to access registered therapy animals and their handlers, and service dogs can come with significant expense. Furthermore, interventions that include pet dogs may be more sustainable and generalizable than those with a registered therapy dog since the pet dog, as a key intervention component, is not removed from the child's environment at the end of therapy. Importantly, pre-existing human-animal bonds may be further strengthened, and novelty is removed as a potential motivator as the pet dog is ever-present in the home.

Pet dogs can provide “in-house” vehicles and prompts for the promotion of physical activity and positive nutrition behaviors in a number of ways. For children who may avoid physical activity because of gross-motor skill deficits, a pet dog does not judge or grow impatient when a child evidences these challenges. Anxiety, a common co-morbidity of ASD, could be mitigated by a program emphasizing a pet dog's non-judgmental attitude toward the child. This is consistent with the observation that dogs have a calming effect on children with ASD (22). Similarly, introducing nutrition-related concepts through the lens of the dog's health may serve to increase knowledge and awareness in a less stressful manner because the emphasis is not directed at the child's problematic eating behaviors. Dogs may also provide an indirect approach to modeling healthy eating behaviors without stigma (e.g., it is possible to discuss how overeating and obesity can lead to diseases in dogs without inducing guilt or blame in children). Additionally, planning a shopping list to meet a pet dog's dietary needs (given that many healthy fruits and vegetables can be shared among children and dogs) may be an accessible entrée to learning to plan grocery shopping. Other areas of pet care may provide similarly useful models for self-care. A social story around a pet dog going to a veterinarian for a check-up can help prepare a child for understanding a visit to a medical provider in a setting that is distinct from a child's own potential anxiety around experiences such as being touched by a stranger or receiving a vaccination (23). These areas also provide opportunity to strengthen the bond between the family and the pet dog, while improving the health and well-being of the pet dog by increasing canine health literacy among families and promoting physical activity and proper nutrition for all family members, human and canine alike.

### Assessing the Fit

Several important issues must be considered for any AAI with pet dogs for children with ASD. One key consideration is the relationship between the pet dog and the child. Based on their known characteristics, some dogs may be better suited for this type of intervention than others, and it is important to know the individual animal, their sensitivities, and temperament. Certain breeds (e.g., golden retrievers, Labradors) are commonly trained to be service or therapy dogs; however, many other breeds can enjoy their role as a suitable companion or other helping animal. Family knowledge of the specific dogs and their needs in training is likely to be one of the most important predictors of success. For example, herding breeds that require extensive physical activity and mental enrichment may not be the best fit; however, many Pit Bulls are registered therapy dogs and provide enrichment for their families as well as enjoy the work themselves. Incorporating older dogs who are more mature or waiting to introduce AAI with a pet dog once the relationship within the family dynamic is fully established is also crucial for success. Pet allergies among family members is an additional consideration.

In the context of the family, the pet dog should be socially motivated, healthy, and have basic obedience training. The child should have at least a neutral relationship with the dog, if not a positive one, with no history of negative interactions including physical harm or threat of physical harm to the dog. Importantly, the child should possess some safety awareness both for themselves and for the dog. Viewing AAI as a partnership in which the pet dog benefits from and enjoys interaction just as much as the child and caregivers is critical to successful integration of a dog “with a job” into the family unit. Families who have not had a dog in their households previously should be realistic about the care requirements of a pet dog as well as the financial resources required. Costs include food and treats, clothing for harsh climates, grooming, walking/boarding, training if a younger dog, and veterinary expenses including preventive healthcare. The authors advise consultation with experts and careful consideration for the well-being and best fit for all family members, especially the pet dog, before adoption occurs. Although pet dogs can provide many benefits, they have substantial needs of their own, including daily care, attention, veterinary healthcare and associated costs. Additionally, given the demands of raising child with ASD, adding another dependent being may be burdensome. For all of these reasons, decisions to add a pet dog to a family unit should be carefully considered.

### Beyond Obesity

The potential of an animal-assisted approach for building skills that positively impact health are numerous. These include, but are not limited to, aspects of self-care, establishing healthy routines, and sleep hygiene. For example, many individuals with ASD have limitations in their ability to function independently and require assistance to master self-care skills (24, 25). These deficits can include daily skills such as hygiene, food preparation, and other health-promoting behaviors; food purchasing skills are also an area of specific concern (26).

### Research Needs and Considerations

Several avenues of future studies are suggested to realize the potential of AAI with pet dogs to promote healthy weight in children with ASD. Formative research is needed to investigate who and how best to deliver an AAI with pet dogs. Possibilities include parents and specialists already working with children with ASD, including applied behavioral analysts or occupational therapists. Formative research could provide insights into the existing relationships between children with ASD and their pet dogs, as well as how physical activity and nutrition promotion might be integrated within family life in households that include a pet dog. With greater understanding of the child with ASD and the pet-owning family, interventions to promote healthy behaviors and meet the particular needs of this population while ensuring safety and well-being of the canine participants can be designed and evaluated.

Guidelines for the design of psychosocial intervention research for individuals with ASD (27) are applicable to trials aimed at health promotion through use of the pet dog. This phased “model” is non-prescriptive but starts with formulation of a new intervention technique and single-case or small pilot designs. If initial feasibility and efficacy is established, the final phases call for randomized clinical trials and community effectiveness studies (27). Relevant short-term outcomes would include engagement, participation, and enjoyment; medium-term outcomes would include improvements in physical activity and eating behaviors. Longer term outcomes would include decreased risk of obesity and obesity-related chronic diseases, cost benefits, and optimal opportunities for independent living.

Finally, in preparing this opinion we seek to start a dialog, and invite experts in a range of disciplines, including special education, adaptive physical education, developmental and behavioral pediatrics, occupational therapy, veterinary medicine, and nutrition to share their perspectives on the opportunity to involve pet dogs in promoting healthy weight and wellness for youth with ASD.

## Author Contributions

AM, CM, DL, SC, and SF: conceptualization, writing – original draft preparation, writing – review, and editing. All authors contributed to the article and approved the submitted version.

## Conflict of Interest

DL was a faculty advisor for a Pet Partner community partner group at Tufts University. The funders had no role in the design of the study; in the collection, analyses, or interpretation of data; in the writing of the manuscript, or in the decision to publish the results. The remaining authors declare that the research was conducted in the absence of any commercial or financial relationships that could be construed as a potential conflict of interest.
